# Letter to the Editor From Tapia-Castillo et al.: “Considerations About the Indirect Role of Low Cortisone in Subjects With Normal Cortisol to Cortisone Ratio”

**DOI:** 10.1210/jendso/bvae138

**Published:** 2024-07-26

**Authors:** Alejandra Tapia-Castillo, Cristian A Carvajal, Carlos E Fardella

**Affiliations:** Department of Endocrinology, School of Medicine, Pontificia Universidad Católica de Chile, Santiago 8330077, Chile; Centro Traslacional de Endocrinología UC (CETREN-UC), Santiago 8330077, Chile; Department of Endocrinology, School of Medicine, Pontificia Universidad Católica de Chile, Santiago 8330077, Chile; Centro Traslacional de Endocrinología UC (CETREN-UC), Santiago 8330077, Chile; Department of Endocrinology, School of Medicine, Pontificia Universidad Católica de Chile, Santiago 8330077, Chile; Centro Traslacional de Endocrinología UC (CETREN-UC), Santiago 8330077, Chile

We appreciate the comments of Armanini et al [1] concerning our recent article published in the *Journal of the Endocrine Society* [2]. We would like to clarify the following concepts.

Armanini et al emphasizes the role that the enzyme 11β-HSD1 may have in cortisone levels. The 11β-HSD1 enzyme, which converts cortisone to cortisol, is located mainly in the liver and adipose tissue; the cortisone when it passes through the liver to the systemic circulation is converted into tetrahydrocortisone [3].

Human studies have suggested that an overexpression of 11β-HSD1 in subcutaneous abdominal adipose tissue induces increased production of local cortisol [4]. In this sense, although in our study we observed higher body mass index (BMI) in low E subjects, we did not observe increased serum cortisol levels. However, we did observe a decrease in cortisone levels, along with low renin activity, suggestive of a lower 11β-HSD2 activity.

The other factors cited by Armanini, such as aldosterone, renin, steroid-binding proteins, aldosterone synthase (CYP11B2), do not influence in cortisone level. However, factors such as adrenocorticotropin, NAD cofactor, and endogenous or exogenous metabolites influence the normal functioning of the enzyme 11β-HSD2, and that ultimately lead to less metabolization of cortisol to cortisone.

Another point cited by Armanini and colleagues is that our study reports increased protein expression of plasminogen activator inhibitor-1 (PAI-1) in subjects with low E, which could be explained because our patients have a higher BMI. However, when we performed correlation analysis normalized by BMI, we observed that cortisone is inversely associated with PAI-1; therefore is very unlikely that this profile is due to obesity. Moreover, Calo et al in 2004 [5], observed that higher concentrations of aldosterone were instead able to stimulate the levels of PAI-1 in all subjects, suggesting a role mediated by mineralocorticoid receptor activation. In relation to aldosterone, this work does not ignore the modifications of the inflammatory profile due to a simultaneous primary aldosteronism; in fact our group recently published the coexistence of both diseases [6].

Finally, our findings highlight the concept that the low-renin phenotype is dictated not only by serum aldosterone levels but also by low cortisone. In this regard, we agree that cortisone could be an early marker of arterial hypertension and inflammation in normotensive subjects and could explain an unknown number of cases of low renin hypertension.

## Disclosures

The authors declare that there is no conflict of interest.
